# A population-based study on meteorological conditions in association with motor vehicle collisions among people with type 2 diabetes

**DOI:** 10.1265/ehpm.25-00308

**Published:** 2025-11-19

**Authors:** Chung-Yi Li, Ya-Hui Chang, Hon-Ping Ma, Ping-Ling Chen, Chang-Ta Chiu, I-Lin Hsu

**Affiliations:** 1 Department of Public Health, College of Medicine, National Cheng Kung University, Tainan, Taiwan; 2 Department of Public Health, College of Public Health, China Medical University, Taichung, Taiwan; 3 Department of Healthcare Administration, College of Medical and Health Science, Asia University, Taichung, Taiwan; 4 Emergency Department, Shuang Ho Hospital, Taipei Medical University, New Taipei City, Taiwan; 5 Graduate Institute of Injury Prevention and Control, College of Public Health, Taipei Medical University, Taipei, Taiwan; 6 Department of Emergency, School of Medicine, College of Medicine, Taipei Medical University, Taipei, Taiwan; 7 Department of Dentistry, An Nan Hospital, China Medical University, Tainan, Taiwan; 8 The Cross College Elite Program, National Cheng Kung University, Tainan, Taiwan; 9 Division of Surgery, National Cheng Kung University Hospital, College of Medicine, National Cheng Kung University, Tainan, Taiwan

**Keywords:** Type 2 diabetes, MVC, Temperature, Rainfall, Wind speed, Sunshine duration, DLNM

## Abstract

**Background:**

Prior studies have shown that drivers with type 2 diabetes are more likely to be involved in motor vehicle collisions (MVCs) compared to the general population. Certain meteorological factors have been increasingly recognized as contributors to MVC risk. This study aims to examine the association of MVCs with temperature, rainfall, wind speed, and sunshine duration among drivers with type 2 diabetes.

**Methods:**

Using Taiwan’s National Health Insurance data (2019–2021), we identified individuals diagnosed with type 2 diabetes and linked their records to the Police-Reported Traffic Accident Registry to obtain daily MVC counts. Meteorological data were sourced from the Central Weather Administration. Associations between daily weather conditions and MVCs were assessed using a Distributed Lag Non-Linear Model.

**Results:**

Over the 1,096-day study period, 170,468 MVC events involving drivers with type 2 diabetes were recorded. A U-shaped association was observed between same-day temperature and MVC rates. Compared with the reference temperature of 17.5 °C, both lower temperatures (≤15 °C; rate ratio [RR] = 1.014–1.053) and higher temperatures (≥30 °C; RR = 1.062) were associated with increased MVC risk. Rainfall showed an inverse relationship with MVCs. Compared with 70 mm of rainfall, the lowest MVC rate occurred at 129 mm (RR = 0.873), while the highest was on rain-free days (0 mm; RR = 1.068). Stronger effects were observed when lag periods up to 14 days were considered. Wind speed and sunshine duration were not significantly associated with MVC risk.

**Conclusions:**

These findings suggest that drivers with type 2 diabetes should exercise greater caution on days with extreme temperatures or in days with lesser rainfall, as these conditions may elevate MVC risk.

**Supplementary information:**

The online version contains supplementary material available at https://doi.org/10.1265/ehpm.25-00308.

## Introduction

Type 2 diabetes mellitus is a growing global health concern, with an estimated 537 million adults affected worldwide in 2021, a number projected to rise to 783 million by 2045 [1]. The disease imposes a significant burden due to its complications, including cardiovascular disease, neuropathy, and impaired thermoregulation, which collectively diminish quality of life and increase mortality [2]. In addition to these complications, the heightened risk of motor vehicle collisions (MVCs) in individuals with type 2 diabetes is particularly concerning. Prior studies have shown that drivers with type 2 diabetes are more likely to be involved in MVCs compared to the general population, attributable to factors such as hypoglycemia, cognitive impairment, and delayed reaction times [3, 4].

People with type 2 diabetes are especially vulnerable to environmental stressors, including extreme weather conditions. Impaired skin blood flow (SkBF) and reduced sweating capacity, common in type 2 diabetes, compromise thermoregulation, increasing susceptibility to heat-related illnesses and potentially impairing driving performance [5, 6]. Furthermore, comorbidities such as obesity and cardiovascular disease exacerbate these risks, making type 2 diabetes patients less resilient to temperature extremes [7].

In addition, diabetes is associated with cognitive impairments that may worsen under thermal stress. Experimental studies show that heat exposure slows reaction time, decreases vigilance, and increases driving errors [8, 9], while cold stress impairs attention, memory, and psychomotor performance [10]. These effects are particularly concerning for drivers with type 2 diabetes, who already face increased risks of hypoglycemia-related cognitive dysfunction and delayed response times [3]. Together, these physiological and cognitive vulnerabilities suggest that extreme meteorological conditions may compound driving risks in this high-risk population.

Meteorological factors, including temperature, wind speed, rainfall, and sunshine duration have been increasingly recognized as contributors to MVC risk. High ambient temperatures have been associated with increased MVC incidence, particularly in drivers with performance-related factors such as distraction or fatigue [11]. A time-series analysis in Catalonia, Spain, showed a 2.9% increase in daily MVCs during heat wave days, with a 7.7% increase for crashes involving driver-performance factors such as fatigue and distraction [12]. Similarly, in the United States, a study in New York found that each 1 °C increase above 26.1 °C was associated with a 1.58% rise in MVC risk over a 0∼2-day lag, while cold temperatures (<−4.8 °C) were associated with an even greater cumulative risk over 0–28 days [8]. Rainfall and reduced visibility due to fog or low sunshine duration further elevate MVC risk by impairing road traction and driver visibility [13, 14].

Despite growing evidence that extreme weather conditions influence road traffic safety, two critical gaps remain. First, most prior studies have focused on the general population, with little attention to high-risk groups such as people with type 2 diabetes, who may be more vulnerable due to impaired thermoregulation, comorbidities, and cognitive or motor deficits [5, 15]. Second, limited evidence exists from Taiwan, where unique climatic patterns and traffic environments may modify these associations [16].

To address these gaps, the present study aims to investigate the associations between daily meteorological conditions (temperature, rainfall, wind speed, and sunshine duration) and the risk of MVCs among drivers with type 2 diabetes in Taiwan, using a nationwide, population-based dataset. Specifically, we hypothesize that: (1) Extreme temperatures (both low and high) are associated with elevated MVC risk among drivers with type 2 diabetes. (2) Rainfall, wind speed, and sunshine duration exert additional influences on MVC risk, although the direction and magnitude of these effects may differ. (3) Lagged effects of meteorological exposures contribute to cumulative risks, reflecting delayed or prolonged physiological and behavioral responses. Accordingly, the specific research questions are: (1) How are daily temperature, rainfall, wind speed, and sunshine duration associated with MVC risk among drivers with type 2 diabetes? (2) Do these associations persist or change when lagged effects up to 14 days are considered? and (3) To what extent do different meteorological factors jointly shape the risk profile for MVCs in this vulnerable population?

## Methods

The study proposal was approved by the Institutional Review Board of National Cheng Kung University Hospital (No. B-ER-112-034). Informed consent was waived due to the use of anonymous personal identification numbers. Access to the research data was approved by the Health and Welfare Data Science Center (HWDSC). To guard the data, all data management and statistical analyses were conducted on-site at HWDSC.

### Source of data

Various meteorological factors were used as explanatory variables in this study, including air temperature, rainfall, wind speed, and sunshine duration, obtained from the Central Weather Administration’s open data. Daily records of these factors were retrieved from 560 weather stations across Taiwan between 2019 and 2021.

The patients with type 2 diabetes were ascertained from the National Health Insurance medical claim data for 2019–2021, including Outpatient, Emergency, and Inpatient Claims. The Police-reported Traffic Accident Registry (PTAR) provides information on daily MVC counts from 2019–2021, the outcome variable of this study.

### Measurement of selected meteorological factors

This study uses daily average meteorological data from 560 official and automated weather stations across Taiwan to calculate national averages for temperature, rainfall, wind speed, and sunshine duration. These variables served as exposure indicators for assessing the relationship between meteorological conditions and MVC risk among drivers with type 2 diabetes. The above-mentioned meteorological factors were retrieved from the Open Weather Data supervised by Taiwan Central Weather Administration (https://opendata.cwa.gov.tw/index).

### People living with type 2 diabetes and driver victims of MVCs

**Fig. 1:**
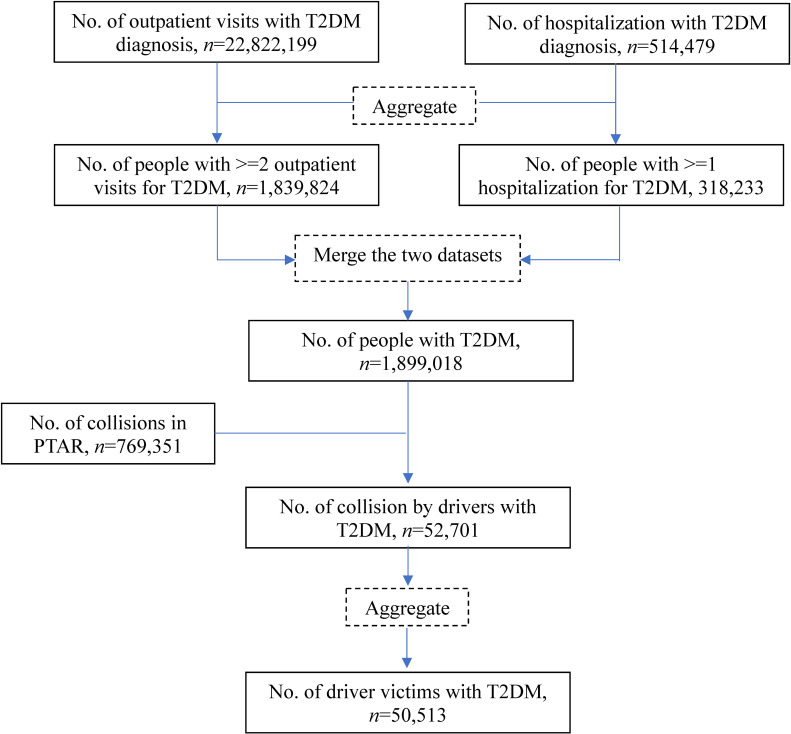
Flow chart of collision case identifications in 2019 T2DM. type 2 diabetes mellitus; PTAR, Police-reported Traffic Accident Registry

We identified people with type 2 diabetes based on the International Classification of Diseases, 10th Revision, Clinical Modification (ICD-10-CM) code E11. Individuals were defined as having type 2 diabetes if they had at least two outpatient/emergency visits or one hospitalization per year [17]. A total of 1,899,018, 1,967,506, and 2,034,278 people with type 2 diabetes were identified from the medical claims of 2019, 2020, and 2021, respectively (Fig. 1 and Figs. S1–S2).

The PTAR includes detailed, standardized records of all police-reported traffic collisions in Taiwan, including the date, time, location, and role of each person involved (driver, passenger, or pedestrian), as well as vehicle type (e.g., car, truck, scooter). These data, based on certified police investigations, have been widely used in traffic injury and public health research in Taiwan [18, 19]. A total of 769,351, 817,349, and 804,368 collisions were registered in the PTAR for 2019, 2020, and 2021, respectively (Fig. 1 and Figs. S1–S2).

We linked people with type 2 diabetes to the PTAR of each year to identify MVCs involving them. The linkage revealed 52,701 collisions involving 50,513 people with type 2 diabetes in 2019. The corresponding numbers for 2020 and 2021 were 57,968 collisions (55,407 people) and 59,799 collisions (57,115 people), respectively (Fig. 1 and Figs. S1–S2).

### Air pollutants as covariates

Fine particulate matter (PM2.5), a key component of air pollution, has been associated with acute impairments in cognitive and cardiovascular functioning, which may elevate MVC risk among older individuals [20, 21]. In addition, nitrogen dioxide (NO_2_) and sulfur dioxide (SO_2_), both byproducts of fossil fuel combustion, have been linked to an increased risk of traffic accidents. Short-term exposure to NO_2_ may impair cognition and cardiovascular function [22], compromising driving performance. Prior research has reported positive associations between NO_2_ and traffic collisions, particularly in high-emission urban areas [23]. A local Taiwanese multi-center study based on 14,973 MVC patients also found associations between MVC rates and both NO_2_ and SO_2_ [24].

A recent study from South Korea reported that ozone (O_3_) exposure was associated with a higher risk of traffic injury [25]. We therefore considered PM_2.5_, NO_2_, SO_2_, and O_3_ as covariates. Information on air pollutant concentrations was provided by the Taiwan Ministry of Environment. The monitoring network includes over 70 fixed-site stations across Taiwan measuring hourly concentrations of ambient pollutants [26]. Daily air pollutant concentrations were represented by the average of 24 hourly measurements.

### Statistical analysis

We applied the Distributed Lag Non-Linear Model (DLNM) to explore relationships between meteorological factors and MVC occurrence among people with type 2 diabetes. The independent variables included the four meteorological factors, with four air pollutant concentrations as covariates. The dependent variable was the daily count of MVCs involving drivers with type 2 diabetes.

As the outcome variable is count data, the DLNM modeled MVC counts using a Poisson distribution, with Y*t* representing MVCs on day *t* (*t* = 1∼1,096). The log(N*ti*) was used as the offset term, where N*ti* represents the number of people with type 2 diabetes on day *t* of year *i* (2019–2021). The number of people with type 2 diabetes was assumed constant within each year *i*. We checked the overdispersion parameter *ψ* (E(Y*i*)/Var(Y*i*)) and found no evidence of overdispersion, as *ψ* did not significantly deviate from 1. The DLNM framework allows simultaneous modeling of non-linear and delayed effects of environmental exposures across a specified lag period [27].

To specify the exposure-response function and lag structure, we compared candidate forms including natural cubic splines, B-splines, polynomial functions, and linear terms, each with 2–5 degrees of freedom. Candidate models were evaluated using the Akaike Information Criterion (AIC) and residual diagnostics to balance goodness-of-fit with parsimony [28]. Both natural cubic splines and third-degree polynomials fit acceptably, but the latter was selected for its slightly lower AIC and better interpretability. For the lag structure, we assumed a linear form across lag days, a common simplification to avoid overfitting and improve interpretability in time-series analyses [27]. We estimated effects for same-day exposures and cumulative lags of 1, 3, 7, and 14 days to capture both immediate and delayed meteorological influences. A sensitivity analysis based on the cumulative lags of 30 days was conducted to assess the potential effects of meteorological conditions on MVCs beyond 14 days.

We first estimated crude rate ratios (RRs) and 95% confidence intervals (CIs) for MVCs in relation to specific meteorological factors. We then fitted DLNMs including all four meteorological factors (temperature, rainfall, wind speed, and sunshine duration) and four air pollutants (PM_2.5_, NO_2_, SO_2_, and O_3_) to estimate adjusted RRs. To assess potential multicollinearity among the independent variables, we calculated the Variance Inflation Factor (VIF) for each predictor. The VIF values ranged from 1.21 (rainfall) to 4.26 (PM_2.5_), indicating low (VIF = 1–2) to moderate (VIF = 2–5) correlations. These results suggest no substantial multicollinearity concerns among the explanatory variables [29, 30]. The All analyses were conducted using the R package *dlnm*, with significance set at α = 0.05.

## Results

Table 1 presents the characteristics of individuals with type 2 diabetes identified between 2019 and 2021. The average age was approximately 64 years, with those aged 65 and older comprising about 50% of the cohort, the largest age group. The proportion of males was slightly higher than that of females. During the study period, approximately 2.7% to 2.8% of individuals with type 2 diabetes were involved in at least one motor vehicle collision (MVC). Both the number of individuals involved in MVCs and the total number of MVC events showed an increasing trend over time.

**Table 1:** Characteristics of people with type 2 diabetes in 2019, 2020, and 2021, respectively.

	**2019**		**2020**		**2021**	
	***n***	**%**	***n***	**%**	***n***	**%**
Total no. of people with type 2 diabetes	1,899,018	100.0	1,967,506	100.0	2,034,278	100.0
Age (years)						
<50	267,585	14.1	276,239	14.04	283,428	13.93
50–64	729,530	38.4	739,363	37.58	746,930	36.72
>=65	901,903	47.5	951,904	48.38	1,003,920	49.35
Mean ± SD	63.9 ± 12.9		64.0 ± 12.9		64.2 ± 12.9	
Sex						
Men	995,891	52.4	1,033,754	52.5	1,071,424	52.7
Women	903,127	47.6	1,020,222	47.5	962,854	47.3
No. of driver victims	50,513	2.7	55,407	2.8	57,115	2.8
No. of MVCs	52,701	-	57,968	-	59,799	-

Daily trends in MVC events and four selected meteorological variables are illustrated in Figs. S3 to S6. MVCs were more frequent during the winter months, particularly in January and February, with daily counts occasionally exceeding 250 cases. As shown in Fig. S3, a sharp decline in MVCs occurred in June 2021, likely due to COVID-19 restrictions, followed by a gradual rebound. Seasonal temperature variation was evident, with the hottest period observed from June to August (average daily temperature around 30 °C) and the coldest in January (around 10 °C).

Rainfall trends (Fig. S4) indicate a primary rainy season from May to September. Peak daily rainfall, often exceeding 120 mm, occurred in May and August, likely due to the plum rain season and typhoons, while most other days recorded less than 10 mm of rain. Wind speed data (Fig. S5) showed that daily averages generally ranged between 1 and 3 m/s, with no clear seasonal pattern. Sunshine duration, shown in Fig. S6, was presented at a monthly resolution, with average daily sunshine duration remaining constant within each month. Nonetheless, seasonal variation was evident, with daily sunshine exceeding 8 hours during summer and dropping to approximately 3 hours in winter.

**Fig. 2:**
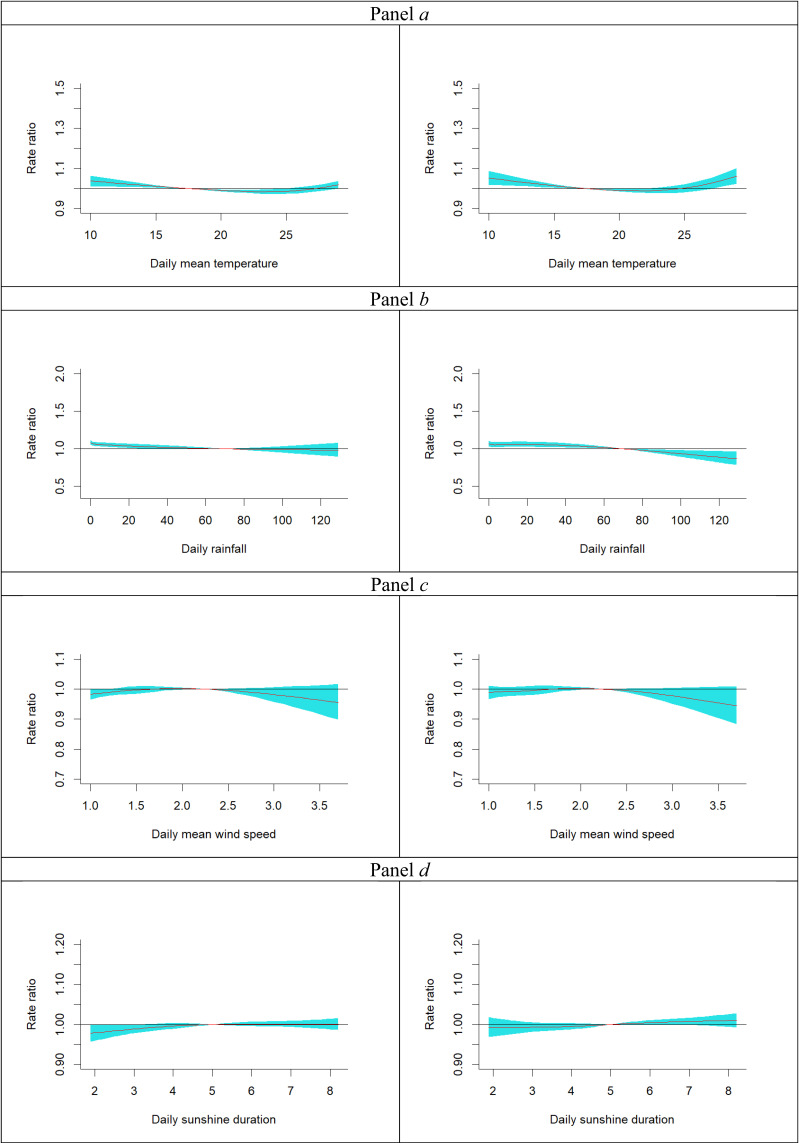
Rate ratios of MVCs in association with same-day exposure to meteorological factors. Temperature (°C): panel *a* left (crude rate ratio) / right (adjusted rate ratio) Rainfall (mm): panel *b* left (crude rate ratio) / right (adjusted rate ratio) Wind speed (m/sec): panel *c* left (crude rate ratio) / right (adjusted rate ratio) Sunshine duration (hours): panel *d* left (crude rate ratio) / right (adjusted rate ratio): The shadow region indicates 95% confidence interval.

Associations between meteorological factors and the rate ratio (RR) of MVCs are presented in Fig. 2. In Panel *a* (left), the unadjusted model revealed an inverse association between same-day temperature and MVC risk. However, the adjusted model in Panel *a* (right), which accounted for the other three meteorological factors and four air pollutants, showed that RRs continued to decrease when temperatures were below 24 °C but began to increase when temperatures exceeded this threshold.

Compared to the reference same-day temperature of 17.5 °C, significantly elevated RRs were observed at temperatures below 15.0 °C (RR = 1.014–1.053) and at 30 °C (RR = 1.062, 95% CI: 1.024–1.102; see Table 2). The U-shaped association between temperature and MVC risk became more pronounced when lag periods were considered. Figures S7–S10, Panel *a*, illustrate these associations across 1-day, 3-day, 7-day, and 14-day lag periods. Notably, the highest RRs associated with an average temperature of 30 °C over these lag periods progressively increased to 1.122 (95% CI: 1.047–1.203), 1.234 (95% CI: 1.092–1.395), 1.404 (95% CI: 1.169–1.687), and 1.445 (95% CI: 1.147–1.820), respectively (see Tables S1–S4).

**Table 2:** Rate ratios of MVCs in association with various same-day 24-hour average temperature.

**Temperature (°C)**	**Model 1** **Unadjusted ** **RR (95% CI)^b^**	**Model 2** **Meteorological and air ** **pollutants adjusted^a^** **RR (95% CI)^b^**
Temperature associated with the lowest RR		
22		0.990 (0.979–1.001)
23	0.985 (0.975–0.995)	
Temperature associated with the highest RR		
10	1.038 (1.012–1.065)	
29		1.062 (1.024–1.102)
Gradient relationship between temperature and RR		
10	1.038 (1.012–1.065)	**1.053 (1.019–1.088)**
15	1.011 (1.005–1.018)	**1.014 (1.006–1.022)**
20	0.991 (0.987–0.995)	0.992 (0.986–0.997)
25	0.987 (0.973–1.001)	1.002 (0.981–1.023)
30	1.019 (1.000–1.038)	**1.062 (1.024–1.102)**

RR, rate ratio; CI, confidence interval

^a^Meteorological factors include rainfall, wind speed, and sunshine duration and air pollutants include PM_2.5_, CO, SO_2_, and O_3_.

^b^Reference temperature: 17.5 °C.

Regarding rainfall, Fig. 2, Panel *b* (left) shows little association with MVCs in the unadjusted model. However, the adjusted model, Panel *b* (right) revealed a clear inverse linear relationship, heavier rainfall was associated with a lower relative risk of MVCs. Compared to the average daily rainfall of 70 mm, the highest RR was observed on days without rain (0 mm; RR = 1.068, 95% CI: 1.031–1.107), while the lowest RR occurred on days with 125 mm of rain (RR = 0.882, 95% CI: 0.803–0.968; see Table 3). This inverse association became more prominent with longer lag periods (see Panels *b* of Figs. S7–S10 and Tables S5–S8).

**Table 3:** Rate ratios of MVCs in association with various same-day rainfall.

**Rainfall (mm)**	**Model 1** **Unadjusted ** **RR (95% CI)^b^**	**Model 2** **Meteorological and air ** **pollutants adjusted^a^** **RR (95% CI)^b^**
Rainfall associated with the lowest RR		
129	0.982 (0.895–1.078)	0.873 (0.789–0.966)
Rainfall associated with the highest RR		
0	1.084 (1.057–1.113)	1.068 (1.031–1.107)
Gradient relationship between rainfall and RR		
0	1.084 (1.057–1.113)	1.068 (1.031–1.107)
25	1.033 (1.004–1.062)	1.055 (1.022–1.089)
50	1.011 (0.992–1.031)	1.032 (1.011–1.054)
75	0.998 (0.992–1.004)	**0.991 (0.984–0.997)**
100	0.989 (0.948–1.032)	**0.938 (0.895–0.983)**
125	0.983 (0.902–1.071)	**0.882 (0.803–0.968)**

RR, rate ratio; CI, confidence interval

^a^Meteorological factors include temperature, wind speed, and sunshine duration and air pollutants include PM_2.5_, CO, SO_2_, and O_3_.

^b^Reference rainfall: 70 mm.

No significant associations were observed between same-day wind speed (Fig. 2, Panels *c*; Table 4) or sunshine duration (Fig. 2, Panels *d*; Table 5) and MVC risk. These null findings persisted regardless of the lag period considered. For detailed results, refer to Panels *c* (wind speed) and *d* (sunshine duration) of Figs. S7–S10, and Tables S9–S12 (wind speed) and Tables S13–S16 (sunshine duration).

**Table 4:** Rate ratios of MVCs in association with various same-day 24-hour average wind speed.

**Wind speed** **(meter/second, m/s)**	**Model 1** **Unadjusted ** **RR (95% CI)^b^**	**Model 2** **Meteorological and air ** **pollutants adjusted^a^** **RR (95% CI)^b^**
Wind speed associated with the lowest RR		
3.7	0.956 (0.899–1.017)	0.945 (0.884–1.010)
Wind speed associated with the highest RR		
2.0	1.002 (0.998–1.005)	1.002 (0.998–1.007)
Gradient relationship between wind speed and RR		
1.0	0.984 (0.965–1.002)	0.989 (0.968–1.012)
1.7	1.000 (0.991–1.009)	1.000 (0.989–1.011)
2.4	0.998 (0.995–1.001)	0.997 (0.994–1.001)
3.1	0.979 (0.950–1.007)	0.973 (0.943–1.005)
3.7	0.956 (0.899–1.017)	0.945 (0.884–1.010)

RR, rate ratio; CI, confidence interval

^a^Meteorological factors include temperature, rainfall, and sunshine duration and air pollutants include PM_2.5_, CO, SO_2_, and O_3_.

^b^Reference wind speed: 2.25 m/s.

**Table 5:** Rate ratios of MVCs in association with various same-day sunshine duration.

**Sunshine duration ** **(hours)**	**Model 1** **Unadjusted ** **RR (95% CI)^b^**	**Model 2** **Meteorological and air ** **pollutants adjusted^a^** **RR (95% CI)^b^**
Sunshine hours associated with the lowest RR		
2	0.979 (0.959–0.999)	0.993 (0.970–1.017)
3		
Sunshine hours associated with the highest RR		
7	1.001 (0.994–1.009)	
8		1.010 (0.994–1.026)
Gradient relationship between sunshine hours and RR		
2	0.979 (0.959–0.999)	0.993 (0.970–1.017)
4	0.996 (0.989–1.003)	0.995 (0.988–1.003)
6	1.001 (0.996–1.007)	1.005 (0.999–1.011)
8	1.001 (0.988–1.014)	1.010 (0.994–1.026)

RR, rate ratio; CI, confidence interval

^a^Meteorological factors include temperature, rainfall, wind speed and air pollutants include PM_2.5_, CO, SO_2_, and O_3_.

^b^Reference sunshine hours: 5 hours.

The sensitivity analysis indicated that although a U-shaped relationship was observed between daily mean temperature and the MVC rate ratio, this association was not statistically significant. The only statistically significant finding was that a higher MVC rate ratio was associated with a daily sunshine duration of eight hours over the 30-day lag period (RR = 1.215, 95% CI: 1.054–1.400).

## Discussion

This study highlights a U-shaped association between ambient temperature and MVCs among drivers with type 2 diabetes. It reveals that extreme temperatures—both low and high—significantly elevate MVC risk, whereas rainfall has a modest or inverse impact, and wind speed and sunshine are negligible. These results suggest vulnerabilities among drivers with T2DM linked to thermal stress, physiological sensitivity, and behavioral changes in adverse weather. The findings have substantial implications for personal health management, public health policies, and road safety strategies in Taiwan, especially amid climate change.

The findings of this Taiwan-based study are consistent with and extend previous work linking extreme temperatures to increased MVC risk. A time-series study in Spain found MVC risk rose 2.9% during heatwaves [9]. Similarly, research from Australia and China showed heightened crash risks during both very high and low temperatures [31, 32]. While many studies examined the general population, the present work is novel in focusing on people with type 2 diabetes mellitus, an at-risk group with impaired thermoregulation and cognition. These results align with Naughton et al. [33] and Hajat et al. [34], who noted greater heat vulnerability in chronic disease populations. This study thus adds evidence by pinpointing the temperature-MVC risk relationship among people with type 2 diabetes.

From a physiological standpoint, individuals with diabetes are more susceptible to temperature extremes due to autonomic neuropathy, which impairs vasomotor responses and sweat production, reducing heat dissipation [5]. Cold exposure can worsen peripheral neuropathy, slow reflexes, and impair tactile sensitivity, crucial for safe driving. Conversely, high temperatures can cause dehydration and hyperglycemia, diminishing cognitive alertness, visual acuity, and reaction time. Zhu et al. [32] reported higher road traffic injury rates during heatwaves, especially among outdoor workers exposed to physical stressors. Li et al. [35] found extreme temperatures affected all road users (pedestrians, cyclists, motorcyclists, and drivers), with lag effects suggesting cumulative physiological strain. For people with type 2 diabetes, these stressors are amplified by impaired homeostatic control, making them particularly prone to driving impairments under thermal stress.

Qiao et al. [36] showed that extreme heat and NO_2_ exposure independently and jointly disrupt metabolic, oxidative, inflammatory, and autophagy pathways in a type 3 diabetes mouse model, aggravating diabetic nephropathy. These findings mirror our observed associations between heat, NO_2_, and MVC risk among type 2 diabetes drivers. Together, they suggest that physiological stress from thermal extremes and pollutants may slow reaction time, reduce cognitive or motor function, or increase fatigue, thereby raising crash risk.

Interestingly, rainfall was inversely associated with MVC incidence. This finding, consistent with Saha et al. [13] and a Swedish zero-crossing temperature study [37], likely reflects risk-averse driving behavior such as slower speeds or trip cancellation. Rain can prompt greater driver caution, lowering collision rates despite poorer road conditions. The lack of association between MVCs and wind speed or sunshine duration may relate to Taiwan’s urban infrastructure, which reduces wind effects, and to generally adequate daylight or artificial lighting mitigating solar variation. Thus, temperature extremes appear to exert the most significant influence on driving safety among people with diabetes.

This study benefits from nationwide, population-level data linked to meteorological observations, providing a robust basis for temporal assessment. Applying DLNMs enabled analysis of delayed and cumulative exposure effects, improving precision [27]. Adjustment for air pollution further reduced confounding. Nonetheless, limitations include the absence of individual-level exposure details, such as travel time or air conditioning use. The unit of analysis was the daily aggregate count of MVCs among people with type 2 diabetes, linked to national daily meteorological and air pollution data. Within this ecological time-series design, individual-level covariates (e.g., socioeconomic status, road conditions, medication use, hypoglycemia, or driving behavior) could not be incorporated. Moreover, most such variables were unavailable. We instead controlled for ecological-level confounders by including major air pollutants (PM_2.5_, NO_2_, SO_2_, and O_3_) and meteorological factors in the models. Results may not generalize to countries with different climates, traffic laws, or healthcare systems. Future studies using wearable sensors or vehicle telemetry could better capture individual exposure and refine risk prediction for vulnerable groups such as those with diabetes.

This study contributes in several ways beyond prior research. First, while earlier studies largely examined weather-related traffic risks in the general population, our analysis focused on drivers with type 2 diabetes - a physiologically vulnerable and under-studied group. Second, unlike studies from Western countries, our work used nationwide Taiwanese data, reflecting distinct climatic and traffic contexts and expanding global evidence. Finally, the findings carry interdisciplinary importance: they not only advance understanding of weather-health interactions but also inform traffic safety and climate adaptation policies by showing how vulnerable road users may be better protected amid increasing climate variability.

In conclusion, the U-shaped risk pattern offers crucial insight for weather-based interventions. As climate volatility rises, Taiwan’s integrated healthcare and transport systems can serve as models by combining clinical care, environmental awareness, and smart infrastructure to protect vulnerable drivers with type 2 diabetes.

## Abbreviations

MVC: motor vehicle collisions
SkBF: skin blood flow
HWDSC: Health and Welfare Data Science Center
PTAR: Police-reported Traffic Accident Registry
ICD-10-CM: International Classification of Diseases, 10th Revision, Clinical Modification
PM_2.5_: Fine particulate matter
NO_2_: nitrogen dioxide
SO_2_: sulfur dioxide
O_3_: Ozone
DLNM: Distributed Lag Non-Linear Model
AIC: Akaike information criterion
RR: rate ratios

## Supplementary information

**Additional file 1:** **Figure S1.** Flow chart of collision case identifications in 2020.

**Additional file 2:** **Figure S2.** Flow chart of collision case identifications in 2021.

**Additional file 3:** **Figure S3.** Temporal relationship between daily mean temperature and daily number of type 2 diabetes driver’s MVC.

**Additional file 4:** **Figure S4.** Temporal relationship between daily rainfall and daily number of type 2 diabetes driver’s MVC.

**Additional file 5:** **Figure S5.** Temporal relationship between daily mean wind speed and daily number of type 2 diabetes driver’s MVC.

**Additional file 6:** **Figure S6.** Temporal relationship between daily sunshine duration and daily number of type 2 diabetes driver’s MVC.

**Additional file 7:** **Figure S7.** Rate ratios of MVCs in association with various meteorological factors over a 1-day lag period.

**Additional file 8:** **Figure S8.** Rate ratios of MVCs in association with various meteorological factors over a 3-day lag period.

**Additional file 9:** **Figure S9.** Rate ratios of MVCs in association with various meteorological factors over a 7-day lag period.

**Additional file 10:** **Figure S10.** Rate ratios of MVCs in association with various meteorological factors over a 14-day lag period.

**Additional file 11:** **Table S1.** Rate ratios of MVCs in association with various averaged temperature over a 1-day lag period.

**Additional file 12:** **Table S2.** Rate ratios of MVCs in association with various averaged temperature over a 3-day lag period.

**Additional file 13:** **Table S3.** Rate ratios of MVCs in association with various averaged temperature over a 7-day lag period.

**Additional file 14:** **Table S4.** Rate ratios of MVCs in association with various averaged temperature over a 14-day lag period.

**Additional file 15:** **Table S5.** Rate ratios of MVCs in association with various averaged rainfall over a 1-day lag period.

**Additional file 16:** **Table S6.** Rate ratios of MVCs in association with various averaged rainfall over a 3-day lag period.

**Additional file 17:** **Table S7.** Rate ratios of MVCs in association with various averaged rainfall over a 7-day lag period.

**Additional file 18:** **Table S8.** Rate ratios of MVCs in association with various averaged rainfall over a 14-day lag period.

**Additional file 19:** **Table S9.** Rate ratios of MVCs in association with various averaged wind speed over a 1-day lag period.

**Additional file 20:** **Table S10.** Rate ratios of MVCs in association with various averaged wind speed over a 3-day lag period.

**Additional file 21:** **Table S11.** Rate ratios of MVCs in association with various averaged wind speed over a 7-day lag period.

**Additional file 22:** **Table S12.** Rate ratios of MVCs in association with various averaged wind speed over a 14-day lag period.

**Additional file 23:** **Table S13.** Rate ratios of MVCs in association with various averaged sunshine hours over a 1-day lag period.

**Additional file 24:** **Table S14.** Rate ratios of MVCs in association with various averaged sunshine hours over a 3-day lag period.

**Additional file 25:** **Table S15.** Rate ratios of MVCs in association with various averaged sunshine hours over a 7-day lag period.

**Additional file 26:** **Table S16.** Rate ratios of MVCs in association with various averaged sunshine hours over a 14-day lag period.

## Declarations

### Data availability statement

In accordance with Taiwan government regulations, National Health Insurance claim data are not publicly accessible and may only be analyzed on-site at the Health and Welfare Data Science Center. In contrast, data on ambient meteorological conditions and air pollutants are available from the Taiwan Government Open Data Platform and can be provided upon request to the corresponding author, Dr. I-L. Hsu.
