# Supplementary material for: A population-based study on meteorological conditions in association with motor vehicle collisions among people with type 2 diabetes
Source: Environ Health Prev Med. 2025 Nov 19;30:91. doi: 10.1265/ehpm.25-00308 (PMC12665916; doi:10.1265/ehpm.25-00308)
Supplement: Supplementary file 1 — Additional file 1: Figure S1. Flow chart of collision case identifications in 2020. [file ehpm-30-091-s001.docx]

No. of hospitalization with T2DM diagnosis, *n*=495,981

No. of outpatient visits with T2DM diagnosis, *n*=23,139,351

Aggregate

No. of people with >=1 hospitalization for T2DM, 307,254

No. of people with >=2 outpatient visits for T2DM, *n*=1,910,142

Merge the two datasets

No. of people with T2DM, *n*=1,967,506

No. of collisions in PTAR, *n*=817,349

No. of collision by drivers with T2DM, *n*=57,968

Aggregate

No. of driver victims with T2DM, *n*=55,407

Figure S1. Flow chart of collision case identifications in 2020.

T2DM. type 2 diabetes mellitus; PTAR, Police-reported Traffic Accident Registry
