# Supplementary material for: A population-based study on meteorological conditions in association with motor vehicle collisions among people with type 2 diabetes
Source: Environ Health Prev Med. 2025 Nov 19;30:91. doi: 10.1265/ehpm.25-00308 (PMC12665916; doi:10.1265/ehpm.25-00308)
Supplement: Supplementary file 4 — Additional file 4: Figure S4. Temporal relationship between daily rainfall and daily number of type 2 diabetes driver’s MVC. [file ehpm-30-091-s004.docx]

Figure S4. Temporal relationship between daily rainfall and daily number of type 2 diabetes driver’s MVC
