# Supplementary material for: A population-based study on meteorological conditions in association with motor vehicle collisions among people with type 2 diabetes
Source: Environ Health Prev Med. 2025 Nov 19;30:91. doi: 10.1265/ehpm.25-00308 (PMC12665916; doi:10.1265/ehpm.25-00308)
Supplement: Supplementary file 9 — Additional file 9: Figure S9. Rate ratios of MVCs in association with various meteorological factors over a 7-day lag period. [file ehpm-30-091-s009.docx]

| Panel *a* | |
| --- | --- |
| 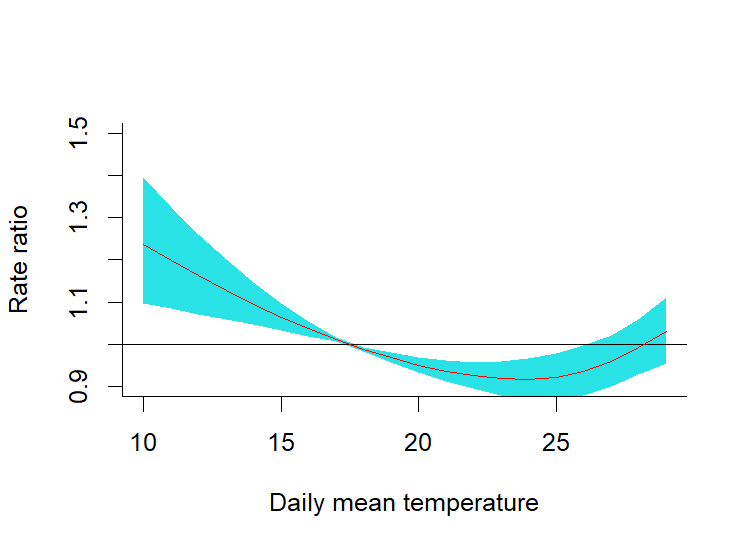 | 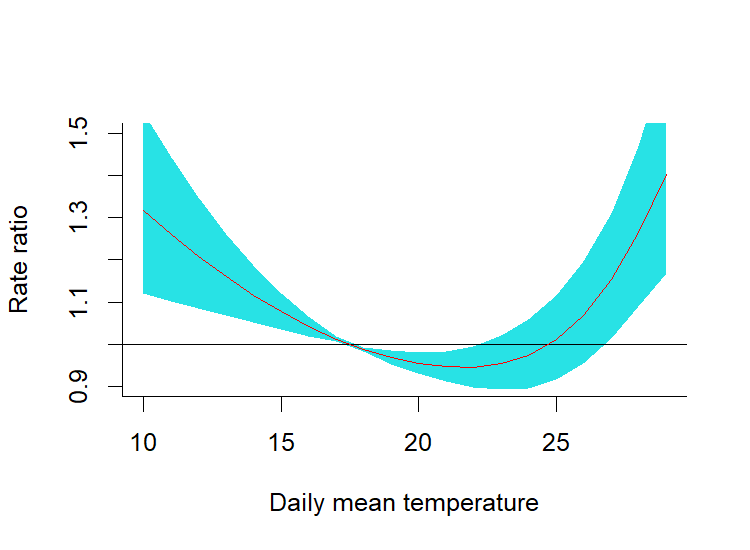 |
| Panel *b* | |
| 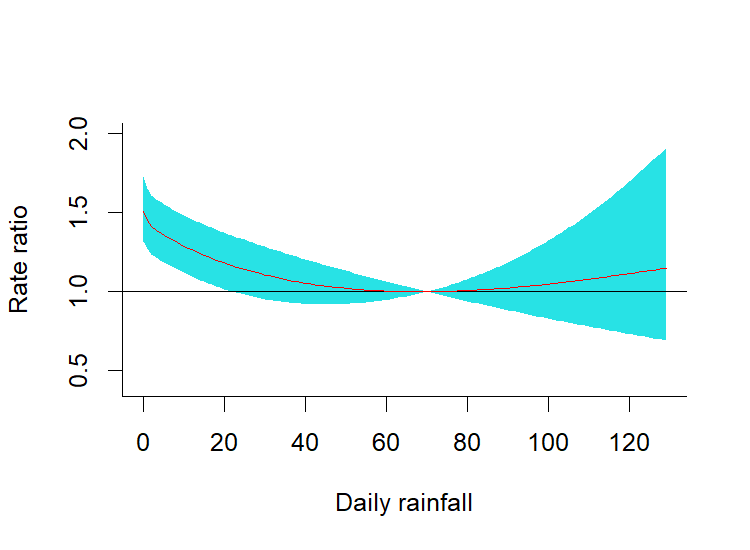 | 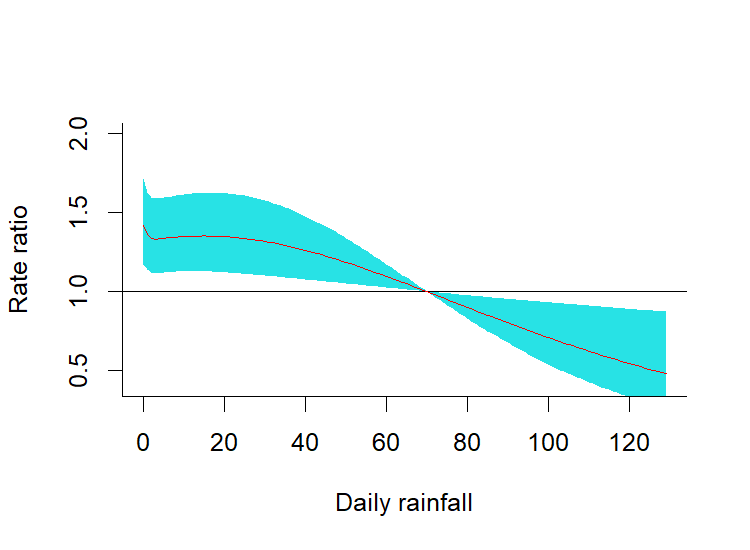 |
| Panel *c* | |
| 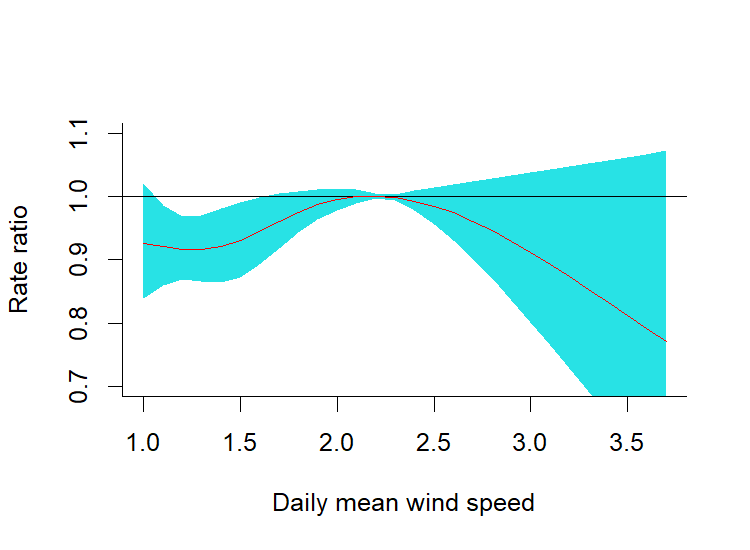 | 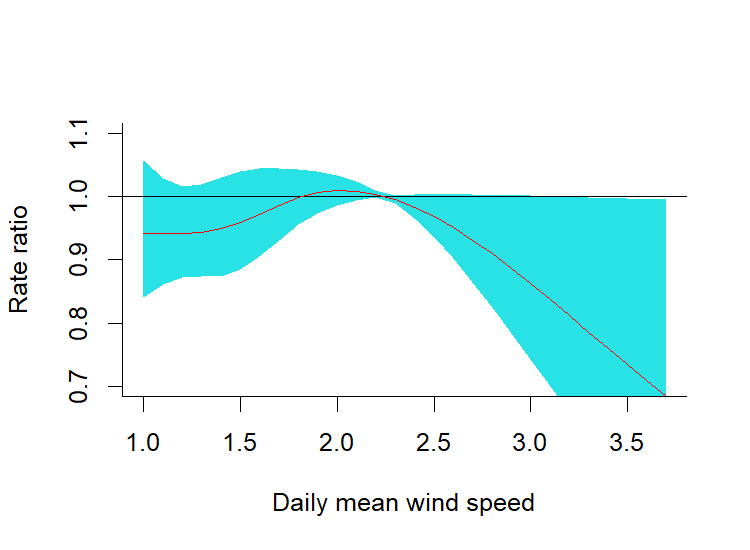 |
| Panel *d* | |
| 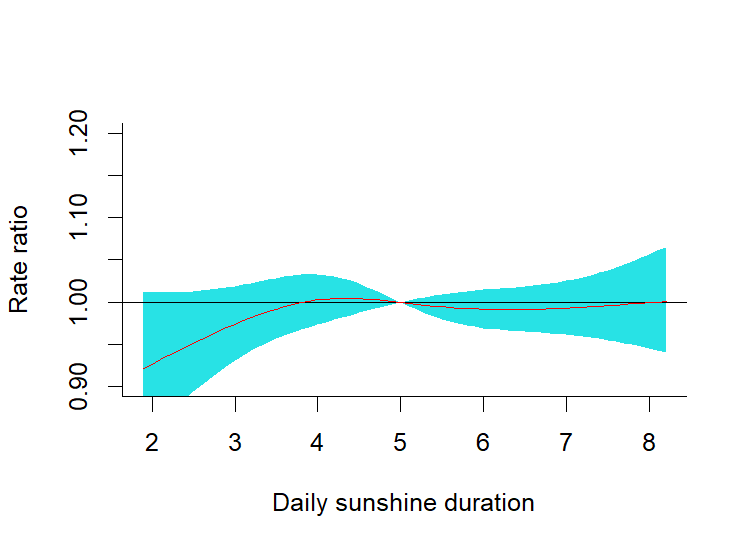 | 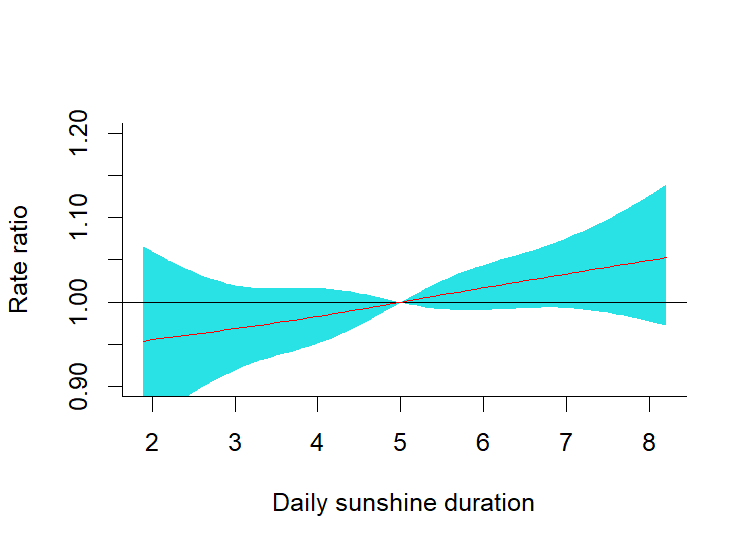 |

Fig S9. Rate ratios of type 2 diabetes drivers’ collisions in association with the **7-day lag period** exposure to various meteorological factors

Temperature (°C): panel *a* left (crude rate ratio) / right (adjusted rate ratio)

Rainfall (mm): panel *b* left (crude rate ratio) / right (adjusted rate ratio)

Wind speed (m/sec): panel *c* left (crude rate ratio) / right (adjusted rate ratio)

Sunshine duration (hours): panel *d* left (crude rate ratio) / right (adjusted rate ratio):

The shadow region indicates 95% confidence interval.
