# Supplementary material for: A population-based study on meteorological conditions in association with motor vehicle collisions among people with type 2 diabetes
Source: Environ Health Prev Med. 2025 Nov 19;30:91. doi: 10.1265/ehpm.25-00308 (PMC12665916; doi:10.1265/ehpm.25-00308)
Supplement: Supplementary file 11 — Additional file 11: Table S1. Rate ratios of MVCs in association with various averaged temperature over a 1-day lag period. [file ehpm-30-091-s011.docx]

Table S1. Rate ratios of MVCs in association with various **averaged temperature over a 1-day lag period**.

| Temperature (℃) | Model 1  Unadjusted  RR (95% CI) ^b^ | Model 2  Meteorological and air pollutants adjusted ^a^  RR (95% CI) ^b^ |
| --- | --- | --- |
| Temperature associated with the lowest RR |  |  |
| 22 |  | 0.981 (0.961-1.001) |
| 23 | 0.971 (0.953-0.990) |  |
| Temperature associated with the highest RR |  |  |
| 10 | 1.074 (1.024-1.126) |  |
| 29 |  | 1.122 (1.047-1.203) |
| Gradient relationship between temperature and RR |  |  |
| 10 | 1.074 (1.024-1.126) | **1.103 (1.037-1.173)** |
| 15 | 1.022 (1.009-1.034) | **1.027 (1.011-1.043)** |
| 20 | 0.983 (0.975-0.991) | 0.984 (0.974-0.994) |
| 25 | 0.975 (0.950-1.001) | 1.004 (0.965-1.044) |
| 30 | 1.033 (0.998-1.070) | **1.122 (1.047-1.203)** |

RR, rate ratio; CI, confidence interval

^a^ Meteorological factors include wind speed, rainfall, and sunshine hours and air pollutants include PM_2.5_, CO, and SO_2_.

^b^ Reference temperature: 17.5 ℃.
