# Supplementary material for: A population-based study on meteorological conditions in association with motor vehicle collisions among people with type 2 diabetes
Source: Environ Health Prev Med. 2025 Nov 19;30:91. doi: 10.1265/ehpm.25-00308 (PMC12665916; doi:10.1265/ehpm.25-00308)
Supplement: Supplementary file 12 — Additional file 12: Table S2. Rate ratios of MVCs in association with various averaged temperature over a 3-day lag period. [file ehpm-30-091-s012.docx]

Table S2. Rate ratios of MVCs in association with various **averaged temperature over a 3-day lag period**.

| Temperature (℃) | Model 1  Unadjusted  RR (95% CI) ^b^ | Model 2  Meteorological and air pollutants adjusted ^a^  RR (95% CI) ^b^ |
| --- | --- | --- |
| Temperature associated with the lowest RR |  |  |
| 22 |  | 0.966 (0.932-1.001) |
| 23 | 0.948 (0.918-0.979) |  |
| Temperature associated with the highest RR |  |  |
| 10 | 1.139 (1.049-1.238) |  |
| 29 |  | 1.234 (1.092-1.395) |
| Gradient relationship between temperature and RR |  |  |
| 10 | 1.139 (1.049-1.238) | **1.193 (1.071-1.329)** |
| 15 | 1.040 (1.018-1.062) | **1.049 (1.021-1.077)** |
| 20 | 0.969 (0.956-0.983) | 0.971 (0.954-0.989) |
| 25 | 0.954 (0.913-0.997) | **1.007 (0.940-1.078)** |
| 30 | 1.050 (0.990-1.113) | **1.234 (1.092-1.395)** |

RR, rate ratio; CI, confidence interval

^a^ Meteorological factors include wind speed, rainfall, and sunshine hours and air pollutants include PM_2.5_, CO, and SO_2_.

^b^ Reference temperature: 17.5 ℃.
