# Supplementary material for: A population-based study on meteorological conditions in association with motor vehicle collisions among people with type 2 diabetes
Source: Environ Health Prev Med. 2025 Nov 19;30:91. doi: 10.1265/ehpm.25-00308 (PMC12665916; doi:10.1265/ehpm.25-00308)
Supplement: Supplementary file 13 — Additional file 13: Table S3. Rate ratios of MVCs in association with various averaged temperature over a 7-day lag period. [file ehpm-30-091-s013.docx]

Table S3. Rate ratios of MVCs in association with various **averaged temperature over a 7-day lag period**.

| Temperature (℃) | Model 1  Unadjusted  RR (95% CI) ^b^ | Model 2  Meteorological and air pollutants adjusted ^a^  RR (95% CI) ^b^ |
| --- | --- | --- |
| Temperature associated with the lowest RR |  |  |
| 22 |  | 0.948 (0.901-0.997) |
| 24 | 0.918 (0.871-0.967) |  |
| Temperature associated with the highest RR |  |  |
| 10 | 1.237 (1.097-1.395) |  |
| 29 |  | 1.404 (1.169-1.687) |
| Gradient relationship between temperature and RR |  |  |
| 10 | 1.237 (1.097-1.395) | **1.317 (1.121-1.548)** |
| 15 | 1.065 (1.033-1.098) | **1.077 (1.035-1.121)** |
| 20 | 0.951 (0.934-0.970) | 0.956 (0.932-0.981) |
| 25 | 0.923 (0.871-0.979) | 1.014 (0.919-1.120) |
| 30 | 1.030 (0.954-1.112) | **1.404 (1.169-1.687)** |

RR, rate ratio; CI, confidence interval

^a^ Meteorological factors include wind speed, rainfall, and sunshine hours and air pollutants include PM_2.5_, CO, and SO_2_.

^b^ Reference temperature: 17.5 ℃.
