# Supplementary material for: A population-based study on meteorological conditions in association with motor vehicle collisions among people with type 2 diabetes
Source: Environ Health Prev Med. 2025 Nov 19;30:91. doi: 10.1265/ehpm.25-00308 (PMC12665916; doi:10.1265/ehpm.25-00308)
Supplement: Supplementary file 14 — Additional file 14: Table S4. Rate ratios of MVCs in association with various averaged temperature over a 14-day lag period. [file ehpm-30-091-s014.docx]

Table S4. Rate ratios of MVCs in association with various **averaged temperature over a 14-day lag period**.

| Temperature (℃) | Model 1  Unadjusted  RR (95% CI) ^b^ | Model 2  Meteorological and air pollutants adjusted ^a^  RR (95% CI) ^b^ |
| --- | --- | --- |
| Temperature associated with the lowest RR |  |  |
| 22 |  | 0.953 (0.915-0.993) |
| 29 | 0.848 (0.813-0.885) |  |
| Temperature associated with the highest RR |  |  |
| 10 | 1.272 (1.108-1.461) |  |
| 29 |  | 1.445 (1.147-1.820) |
| Gradient relationship between temperature and RR |  |  |
| 10 | 1.272 (1.108-1.461) | **1.300 (1.063-1.589)** |
| 15 | 1.072 (1.036-1.109) | **1.073 (1.022-1.126)** |
| 20 | 0.949 (0.933-0.966) | 0.960 (0.932-0.988) |
| 25 | 0.901 (0.865-0.939) | 1.027 (0.915-1.153) |
| 30 | 0.848 (0.813-0.885) | **1.445 (1.147-1.820)** |

RR, rate ratio; CI, confidence interval

^a^ Meteorological factors include wind speed, rainfall, and sunshine hours and air pollutants include PM_2.5_, CO, and SO_2_.

^c^ Reference temperature: 17.5 ℃.
