# Supplementary material for: A population-based study on meteorological conditions in association with motor vehicle collisions among people with type 2 diabetes
Source: Environ Health Prev Med. 2025 Nov 19;30:91. doi: 10.1265/ehpm.25-00308 (PMC12665916; doi:10.1265/ehpm.25-00308)
Supplement: Supplementary file 15 — Additional file 15: Table S5. Rate ratios of MVCs in association with various averaged rainfall over a 1-day lag period. [file ehpm-30-091-s015.docx]

Table S5. Rate ratios of MVCs in association with various **averaged rainfall over a 1-day lag period.**

| Rainfall (mm) | Model 1  Unadjusted  RR (95% CI) ^b^ | Model 2  Meteorological and air pollutants adjusted ^a^  RR (95% CI) ^b^ |
| --- | --- | --- |
| Rainfall associated with the lowest RR |  |  |
| 129 | 0.975 (0.817-1.163) | 0.773 (0.637-0.938) |
| Rainfall associated with the highest RR |  |  |
| 0 | 1.166 (1.110-1.225) | 1.133 (1.060-1.212) |
| Gradient relationship between rainfall and RR |  |  |
| 0 | 1.166 (1.110-1.225) | 1.133 (1.060-1.212) |
| 25 | 1.062 (1.007-1.119) | 1.107 (1.042-1.176) |
| 50 | 1.020 (0.984-1.058) | 1.062 (1.021-1.105) |
| 75 | 0.996 (0.985-1.008) | **0.982 (0.970-0.995)** |
| 100 | 0.983 (0.906-1.066) | **0.886 (0.810-0.968)** |
| 125 | 0.976 (0.829-1.149) | **0.788 (0.659-0.942)** |

RR, rate ratio; CI, confidence interval

^a^ Meteorological factors include wind speed, rainfall, and sunshine hours and air pollutants include PM_2.5_, CO, and SO_2_.

^b^ Reference rainfall: 70 mm.
