# Supplementary material for: A population-based study on meteorological conditions in association with motor vehicle collisions among people with type 2 diabetes
Source: Environ Health Prev Med. 2025 Nov 19;30:91. doi: 10.1265/ehpm.25-00308 (PMC12665916; doi:10.1265/ehpm.25-00308)
Supplement: Supplementary file 16 — Additional file 16: Table S6. Rate ratios of MVCs in association with various averaged rainfall over a 3-day lag period. [file ehpm-30-091-s016.docx]

Table S6. Rate ratios of MVCs in association with various **averaged rainfall over a 3-day lag period.**

| Rainfall (mm) | Model 1  Unadjusted  RR (95% CI) ^b^ | Model 2  Meteorological and air pollutants adjusted ^a^  RR (95% CI) ^b^ |
| --- | --- | --- |
| Rainfall associated with the lowest RR |  |  |
| 104 | 0.985 (0.832-1.166) |  |
| 129 |  | 0.630 (0.444-0.896) |
| Rainfall associated with the highest RR |  |  |
| 0 | 1.314 (1.204-1.434) | 1.251 (1.109-1.410) |
| Gradient relationship between rainfall and RR |  |  |
| 0 | 1.314 (1.204-1.434) | 1.251 (1.109-1.410) |
| 25 | 1.107 (1.006-1.217) | 1.200 (1.075-1.340) |
| 50 | 1.031 (0.967-1.099) | 1.114 (1.036-1.197) |
| 75 | 0.995 (0.975-1.016) | **0.968 (0.946-0.991)** |
| 100 | 0.985 (0.851-1.140) | **0.805 (0.684-0.946)** |
| 125 | 0.988 (0.737-1.325) | **0.652 (0.472-0.903)** |

RR, rate ratio; CI, confidence interval

^a^ Meteorological factors include wind speed, rainfall, and sunshine hours and air pollutants include PM_2.5_, CO, and SO_2_.

^b^ Reference rainfall: 70 mm.
