# Supplementary material for: A population-based study on meteorological conditions in association with motor vehicle collisions among people with type 2 diabetes
Source: Environ Health Prev Med. 2025 Nov 19;30:91. doi: 10.1265/ehpm.25-00308 (PMC12665916; doi:10.1265/ehpm.25-00308)
Supplement: Supplementary file 17 — Additional file 17: Table S7. Rate ratios of MVCs in association with various averaged rainfall over a 7-day lag period. [file ehpm-30-091-s017.docx]

Table S7. Rate ratios of MVCs in association with various **averaged rainfall over a 7-day lag period.**

| Rainfall (mm) | Model 1  Unadjusted  RR (95% CI) ^b^ | Model 2  Meteorological and air pollutants adjusted ^a^  RR (95% CI) ^b^ |
| --- | --- | --- |
| Rainfall associated with the lowest RR |  |  |
| 69 | 1.000 (0.994-1.006) |  |
| 129 |  | 0.494 (0.276-0.883) |
| Rainfall associated with the highest RR |  |  |
| 0 | 1.507 (1.316-1.726) | 1.405 (1.162-1.698) |
| Gradient relationship between rainfall and RR |  |  |
| 0 | 1.507 (1.316-1.726) | 1.405 (1.162-1.698) |
| 25 | 1.140 (0.980-1.327) | 1.327 (1.107-1.591) |
| 50 | 1.021 (0.922-1.132) | 1.181 (1.048-1.331) |
| 75 | 1.002 (0.969-1.036) | **0.952 (0.916-0.989)** |
| 100 | 1.047 (0.830-1.321) | **0.717 (0.549-0.937)** |
| 125 | 1.131 (0.709-1.805) | **0.521 (0.305-0.890)** |

RR, rate ratio; CI, confidence interval

^a^ Meteorological factors include wind speed, rainfall, and sunshine hours and air pollutants include PM_2.5_, CO, and SO_2_.

^b^ Reference rainfall: 70 mm.
