# Supplementary material for: A population-based study on meteorological conditions in association with motor vehicle collisions among people with type 2 diabetes
Source: Environ Health Prev Med. 2025 Nov 19;30:91. doi: 10.1265/ehpm.25-00308 (PMC12665916; doi:10.1265/ehpm.25-00308)
Supplement: Supplementary file 18 — Additional file 18: Table S8. Rate ratios of MVCs in association with various averaged rainfall over a 14-day lag period. [file ehpm-30-091-s018.docx]

Table S8. Rate ratios of MVCs in association with various **averaged rainfall over a 14-day lag period.**

| Rainfall (mm) | Model 1  Unadjusted  RR (95% CI) ^b^ | Model 2  Meteorological and air pollutants adjusted ^a^  RR (95% CI) ^b^ |
| --- | --- | --- |
| Rainfall associated with the lowest RR |  |  |
| 47 | 0.912 (0.776-1.070) |  |
| 129 |  | 0.544 (0.223-1.329) |
| Rainfall associated with the highest RR |  |  |
| 0 |  | 1.331 (1.013-1.750) |
| 129 | 2.180 (1.063-4.473) |  |
| Gradient relationship between rainfall and RR |  |  |
| 0 | 1.380 (1.153-1.650) | 1.331 (1.013-1.750) |
| 25 | 1.011 (0.817-1.249) | 1.299 (0.981-1.719) |
| 50 | 0.913 (0.790-1.056) | 1.160 (0.964-1.396) |
| 75 | 1.043 (0.995-1.093) | **0.957 (0.903-1.016)** |
| 100 | 1.398 (1.005-1.945) | **0.749 (0.496-1.131)** |
| 125 | 2.045 (1.053-3.970) | **0.569 (0.249-1.299)** |

RR, rate ratio; CI, confidence interval

^a^ Meteorological factors include wind speed, rainfall, and sunshine hours and air pollutants include PM_2.5_, CO, and SO_2_.

^b^ Reference rainfall: 70 mm.
