# Supplementary material for: A population-based study on meteorological conditions in association with motor vehicle collisions among people with type 2 diabetes
Source: Environ Health Prev Med. 2025 Nov 19;30:91. doi: 10.1265/ehpm.25-00308 (PMC12665916; doi:10.1265/ehpm.25-00308)
Supplement: Supplementary file 19 — Additional file 19: Table S9. Rate ratios of MVCs in association with various averaged wind speed over a 1-day lag period. [file ehpm-30-091-s019.docx]

Table S9. Rate ratios of MVCs in association with various **averaged wind speed over a 1-day lag period**.

| Wind speed (meter/second, m/s) | Model 1  Unadjusted  RR (95% CI) ^b^ | Model 2  Meteorological and air pollutants adjusted ^a^  RR (95% CI) ^b^ |
| --- | --- | --- |
| Wind speed associated with the lowest RR |  |  |
| 3.7 | 0.917 (0.816-1.031) | 0.896 (0.789-1.017) |
| Wind speed associated with the highest RR |  |  |
| 2.0 | 1.003 (0.996-1.010) | 1.004 (0.996-1.012) |
| Gradient relationship between wind speed and RR |  |  |
| 1.0 | 0.969 (0.935-1.005) | 0.980 (0.939-1.022) |
| 1.7 | 0.999 (0.982-1.016) | 1.000 (0.980-1.020) |
| 2.4 | 0.996 (0.990-1.002) | 0.995 (0.988-1.001) |
| 3.1 | 0.960 (0.908-1.014) | 0.949 (0.893-1.008) |
| 3.7 | 0.917 (0.816-1.031) | 0.896 (0.789-1.017) |

RR, rate ratio; CI, confidence interval

^a^ Meteorological factors include wind speed, rainfall, and sunshine hours and air pollutants include PM_2.5_, CO, and SO_2_.

^b^ Reference wind speed: 2.25 m/s.
