# Supplementary material for: A population-based study on meteorological conditions in association with motor vehicle collisions among people with type 2 diabetes
Source: Environ Health Prev Med. 2025 Nov 19;30:91. doi: 10.1265/ehpm.25-00308 (PMC12665916; doi:10.1265/ehpm.25-00308)
Supplement: Supplementary file 20 — Additional file 20: Table S10. Rate ratios of MVCs in association with various averaged wind speed over a 3-day lag period. [file ehpm-30-091-s020.docx]

Table S10. Rate ratios of MVCs in association with various **averaged wind speed over a 3-day lag period**.

| Wind speed (meter/second, m/s) | Model 1  Unadjusted  RR (95% CI) ^b^ | Model 2  Meteorological and air pollutants adjusted ^a^  RR (95% CI) ^b^ |
| --- | --- | --- |
| Wind speed associated with the lowest RR |  |  |
| 3.7 | 0.854 (0.693-1.051) | 0.812 (0.646-1.020) |
| Wind speed associated with the highest RR |  |  |
| 2.0 | 1.003 (0.992-1.015) | 1.007 (0.992-1.022) |
| Gradient relationship between wind speed and RR |  |  |
| 1.0 | 0.947 (0.889-1.009) | 0.964 (0.894-1.038) |
| 1.7 | 0.992 (0.963-1.022) | 0.998 (0.962-1.034) |
| 2.4 | 0.993 (0.983-1.003) | 0.990 (0.978-1.002) |
| 3.1 | 0.929 (0.842-1.025) | 0.905 (0.812-1.010) |
| 3.7 | 0.854 (0.693-1.051) | 0.812 (0.646-1.020) |

RR, rate ratio; CI, confidence interval

^a^ Meteorological factors include wind speed, rainfall, and sunshine hours and air pollutants include PM_2.5_, CO, and SO_2_.

^b^ Reference wind speed: 2.25 m/s.
