# Supplementary material for: A population-based study on meteorological conditions in association with motor vehicle collisions among people with type 2 diabetes
Source: Environ Health Prev Med. 2025 Nov 19;30:91. doi: 10.1265/ehpm.25-00308 (PMC12665916; doi:10.1265/ehpm.25-00308)
Supplement: Supplementary file 21 — Additional file 21: Table S11. Rate ratios of MVCs in association with various averaged wind speed over a 7-day lag period. [file ehpm-30-091-s021.docx]

Table S11. Rate ratios of MVCs in association with various **averaged wind speed over a 7-day lag period**.

| Wind speed (meter/second, m/s) | Model 1  Unadjusted  RR (95% CI) ^b^ | Model 2  Meteorological and air pollutants adjusted ^a^  RR (95% CI) ^b^ |
| --- | --- | --- |
| Wind speed associated with the lowest RR |  |  |
| 3.7 | 0.772 (0.556-1.072) | 0.690 (0.475-1.003) |
| Wind speed associated with the highest RR |  |  |
| 2.0 |  | 1.010 (0.987-1.034) |
| 2.2 | 1.001 (0.997-1.005) |  |
| Gradient relationship between wind speed and RR |  |  |
| 1.0 | 0.926 (0.840-1.020) | 0.943 (0.841-1.058) |
| 1.7 | 0.960 (0.918-1.004) | 0.988 (0.933-1.047) |
| 2.4 | 0.993 (0.977-1.009) | 0.983 (0.965-1.003) |
| 3.1 | 0.893 (0.765-1.043) | 0.839 (0.703-1.003) |
| 3.7 | 0.772 (0.556-1.072) | 0.690 (0.475-1.003) |

RR, rate ratio; CI, confidence interval

^a^ Meteorological factors include wind speed, rainfall, and sunshine hours and air pollutants include PM_2.5_, CO, and SO_2_.

^b^ Reference wind speed: 2.25 m/s.
