# Supplementary material for: A population-based study on meteorological conditions in association with motor vehicle collisions among people with type 2 diabetes
Source: Environ Health Prev Med. 2025 Nov 19;30:91. doi: 10.1265/ehpm.25-00308 (PMC12665916; doi:10.1265/ehpm.25-00308)
Supplement: Supplementary file 22 — Additional file 22: Table S12. Rate ratios of MVCs in association with various averaged wind speed over a 14-day lag period. [file ehpm-30-091-s022.docx]

Table S12. Rate ratios of MVCs in association with various **averaged wind speed over a 14-day lag period**.

| Wind speed (meter/second, m/s) | Model 1  Unadjusted  RR (95% CI) ^b^ | Model 2  Meteorological and air pollutants adjusted ^a^  RR (95% CI) ^b^ |
| --- | --- | --- |
| Wind speed associated with the lowest RR |  |  |
| 3.7 | 0.744 (0.466-1.188) | 0.580 (0.329-1.025) |
| Wind speed associated with the highest RR |  |  |
| 2.1 |  | 1.008 (0.986-1.029) |
| 2.4 | 1.008 (0.986-1.030) |  |
| Gradient relationship between wind speed and RR |  |  |
| 1.0 | 0.960 (0.842-1.095) | 0.942 (0.809-1.097) |
| 1.7 | 0.854 (0.807-0.904) | 0.956 (0.882-1.038) |
| 2.4 | 1.008 (0.986-1.030) | 0.980 (0.952-1.008) |
| 3.1 | 0.907 (0.728-1.130) | 0.780 (0.595-1.022) |
| 3.7 | 0.744 (0.466-1.188) | 0.580 (0.329-1.025) |

RR, rate ratio; CI, confidence interval

^a^ Meteorological factors include wind speed, rainfall, and sunshine hours and air pollutants include PM_2.5_, CO, and SO_2_.

^b^ Reference wind speed: 2.25 m/s.
