# Supplementary material for: A population-based study on meteorological conditions in association with motor vehicle collisions among people with type 2 diabetes
Source: Environ Health Prev Med. 2025 Nov 19;30:91. doi: 10.1265/ehpm.25-00308 (PMC12665916; doi:10.1265/ehpm.25-00308)
Supplement: Supplementary file 23 — Additional file 23: Table S13. Rate ratios of MVCs in association with various averaged sunshine hours over a 1-day lag period. [file ehpm-30-091-s023.docx]

Table S13. Rate ratios of MVCs in association with various **averaged** **sunshine hours over a 1-day lag period.**

| Temperature (℃) | Model 1  Unadjusted  RR (95% CI) ^b^ | Model 2  Meteorological and air pollutants adjusted ^a^  RR (95% CI) ^b^ |
| --- | --- | --- |
| Sunshine hours associated with the lowest RR |  |  |
| 2 | 0.962 (0.926-0.999) | 0.987 (0.944-1.031) |
| Sunshine hours associated with the highest RR |  |  |
| 7 | 1.002 (0.988-1.016) |  |
| 8 |  | 1.018 (0.989-1.049) |
| Gradient relationship between sunshine hours and RR |  |  |
| 2 | 0.962 (0.926-0.999) | 0.987 (0.944-1.031) |
| 4 | 0.994 (0.981-1.007) | 0.991 (0.977-1.006) |
| 6 | 1.002 (0.992-1.012) | 1.008 (0.997-1.020) |
| 8 | 1.001 (0.977-1.026) | 1.018 (0.989-1.049) |

RR, rate ratio; CI, confidence interval

^a^ Meteorological factors include wind speed, rainfall, and sunshine hours and air pollutants include PM_2.5_, CO, and SO_2_.

^b^ Reference sunshine hours: 5 hours.
