# Supplementary material for: A population-based study on meteorological conditions in association with motor vehicle collisions among people with type 2 diabetes
Source: Environ Health Prev Med. 2025 Nov 19;30:91. doi: 10.1265/ehpm.25-00308 (PMC12665916; doi:10.1265/ehpm.25-00308)
Supplement: Supplementary file 24 — Additional file 24: Table S14. Rate ratios of MVCs in association with various averaged sunshine hours over a 3-day lag period. [file ehpm-30-091-s024.docx]

Table S14. Rate ratios of MVCs in association with various **averaged** **sunshine hours over a 3-day lag period.**

| Temperature (℃) | Model 1  Unadjusted  RR (95% CI) ^b^ | Model 2  Meteorological and air pollutants adjusted ^a^  RR (95% CI) ^b^ |
| --- | --- | --- |
| Sunshine hours associated with the lowest RR |  |  |
| 2 | 0.937 (0.879-1.000) | 0.975 (0.904-1.052) |
| Sunshine hours associated with the highest RR |  |  |
| 8 | 1.002 (0.961-1.044) | 1.033 (0.981-1.087) |
| Gradient relationship between sunshine hours and RR |  |  |
| 2 | 0.937 (0.879-1.000) | 0.975 (0.904-1.052) |
| 4 | 0.992 (0.970-1.014) | 0.986 (0.962-1.010) |
| 6 | 1.001 (0.984-1.018) | 1.014 (0.995-1.033) |
| 8 | 1.002 (0.961-1.044) | 1.033 (0.981-1.087) |

RR, rate ratio; CI, confidence interval

^a^ Meteorological factors include wind speed, rainfall, and sunshine hours and air pollutants include PM_2.5_, CO, and SO_2_.

^b^ Reference sunshine hours: 5 hours.
