# Supplementary material for: A population-based study on meteorological conditions in association with motor vehicle collisions among people with type 2 diabetes
Source: Environ Health Prev Med. 2025 Nov 19;30:91. doi: 10.1265/ehpm.25-00308 (PMC12665916; doi:10.1265/ehpm.25-00308)
Supplement: Supplementary file 25 — Additional file 25: Table S15. Rate ratios of MVCs in association with various averaged sunshine hours over a 7-day lag period. [file ehpm-30-091-s025.docx]

Table S15. Rate ratios of MVCs in association with various **averaged** **sunshine hours over a 7-day lag period.**

| Temperature (℃) | Model 1  Unadjusted  RR (95% CI) ^b^ | Model 2  Meteorological and air pollutants adjusted ^a^  RR (95% CI) ^b^ |
| --- | --- | --- |
| Sunshine hours associated with the lowest RR |  |  |
| 2 | 0.927 (0.850-1.011) | 0.956 (0.861-1.061) |
| Sunshine hours associated with the highest RR |  |  |
| 4 | 1.003 (0.974-1.033) |  |
| 8 |  | 1.048 (0.976-1.125) |
| Gradient relationship between sunshine hours and RR |  |  |
| 2 | 0.927 (0.850-1.011) | 0.956 (0.861-1.061) |
| 4 | 1.003 (0.974-1.033) | 0.984 (0.952-1.017) |
| 6 | 0.992 (0.969-1.015) | 1.017 (0.990-1.043) |
| 8 | 1.000 (0.946-1.057) | 1.048 (0.976-1.125) |

RR, rate ratio; CI, confidence interval

^a^ Meteorological factors include wind speed, rainfall, and sunshine hours and air pollutants include PM_2.5_, CO, and SO_2_.

^b^ Reference sunshine hours: 5 hours.
