# Supplementary material for: A population-based study on meteorological conditions in association with motor vehicle collisions among people with type 2 diabetes
Source: Environ Health Prev Med. 2025 Nov 19;30:91. doi: 10.1265/ehpm.25-00308 (PMC12665916; doi:10.1265/ehpm.25-00308)
Supplement: Supplementary file 26 — Additional file 26: Table S16. Rate ratios of MVCs in association with various averaged sunshine hours over a 14-day lag period. [file ehpm-30-091-s026.docx]

Table S16. Rate ratios of MVCs in association with various **averaged** **sunshine hours over a 14-day lag period.**

| Temperature (℃) | Model 1  Unadjusted  RR (95% CI) ^b^ | Model 2  Meteorological and air pollutants adjusted ^a^  RR (95% CI) ^b^ |
| --- | --- | --- |
| Sunshine hours associated with the lowest RR |  |  |
| 2 |  | 0.937 (0.857-1.024) |
| 6 | 0.951 (0.936-0.967) |  |
| Sunshine hours associated with the highest RR |  |  |
| 3 | 1.073 (1.042-1.105) |  |
| 8 |  | 1.031 (0.960-1.107) |
| Gradient relationship between sunshine hours and RR |  |  |
| 2 | 1.033 (0.969-1.102) | 0.937 (0.857-1.024) |
| 4 | 1.069 (1.047-1.090) | 1.008 (0.982-1.035) |
| 6 | 0.951 (0.936-0.967) | 0.994 (0.971-1.016) |
| 8 | 0.986 (0.947-1.026) | 1.031 (0.960-1.107) |

RR, rate ratio; CI, confidence interval

^a^ Meteorological factors include wind speed, rainfall, and sunshine hours and air pollutants include PM_2.5_, CO, and SO_2_.

^b^ Reference sunshine hours: 5 hours.
